# Idiopathic ascites following laparoscopic appendicectomy: a case report

**DOI:** 10.1093/jscr/rjab454

**Published:** 2021-11-05

**Authors:** Aleksis Martindale, Muhammad Tobbal, Charles Coventry, Darmarajah Veeramootoo

**Affiliations:** Upper Gastrointestinal Surgery Unit, Frimley Park Hospital, Camberley, Surrey GU16 7UJ, UK; Upper Gastrointestinal Surgery Unit, Frimley Park Hospital, Camberley, Surrey GU16 7UJ, UK; Upper Gastrointestinal Surgery Unit, Frimley Park Hospital, Camberley, Surrey GU16 7UJ, UK; Oesophag-Gastric Surgical Unit, Flinders Medical Centre, Bedford Park, South Australia 5042, Australia; Upper Gastrointestinal Surgery Unit, Frimley Park Hospital, Camberley, Surrey GU16 7UJ, UK

## Abstract

Appendicitis is a common condition and is frequently treated with a laparoscopic appendicectomy. We present a rare case of delayed, idiopathic ascites following laparoscopic appendicectomy for histologically confirmed appendicitis. While the complications of this condition and this procedure are well documented, this case demonstrates very rare sequelae following a laparoscopic appendicectomy.

## INTRODUCTION

Appendicitis is a condition commonly encountered by surgeons worldwide and laparoscopic appendicectomy has been performed for this since the 1980’s [1]. There are many well-documented major and minor complications of this procedure. The case presented here describes delayed-onset ascites after a laparoscopic appendicectomy, which has not previously been described, and uses the SCARE criteria to report this [2].

## CASE REPORT

A 47-year-old woman presented to accident and emergency with abdominal pain, distension, reduced appetite and weight loss. She had undergone a laparoscopic appendicectomy for perforated appendicitis 2 months previously, with histology demonstrating appendiceal necrosis with full-thickness acute inflammation. She had seemingly had an uneventful recovery and had been discharged 3 days after the procedure. Her relevant past medical history included an intrauterine device insertion (still *in situ*) and gastro-oesophageal reflux disease medicated with regular omeprazole. Her family history was significant for a maternal grandmother who had uterine cancer. She grew up in Venezuela and had lived in France and the USA before moving to the UK. She denied alcohol use, smoking or other substanceuse.

**Figure 1:**
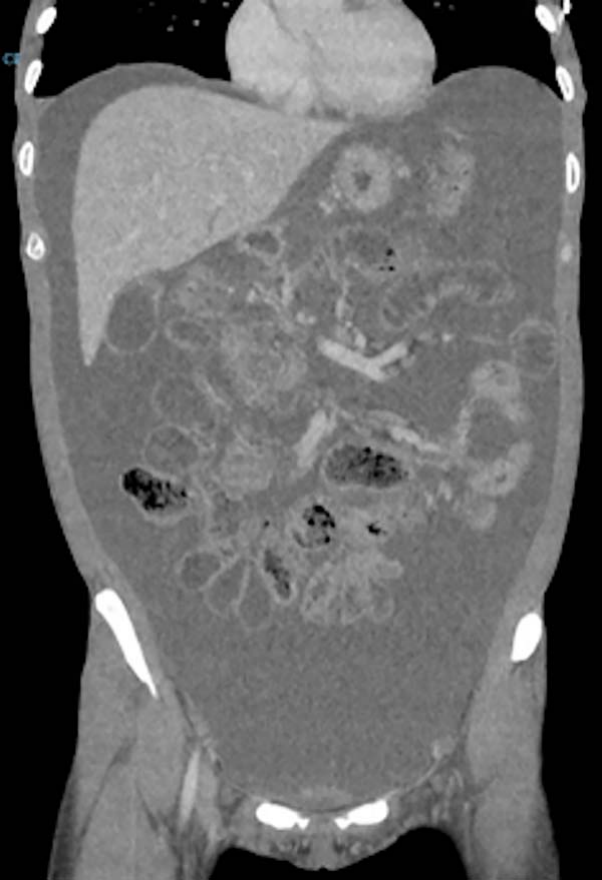
Initial CT scan performed on admission.

On examination, she had tense ascites but no clinical signs of peritonism. Initial blood tests demonstrated normal inflammatory markers, liver function and electrolytes. A computed tomography (CT) scan of the abdomen demonstrated gross ascites, but no defined pathology, including no mass lesions, portal vein thrombosis or peritoneal changes (Fig. 1). An ascitic drain was inserted and drained 4.6 litres of clear fluid. This was sent for cytology, biochemistry and culture (including for tuberculosis), with the results indicated in Table 1. Tumour markers (CEA, CA125, CA19-9 and AFP) were all within normal limits.

**Table 1:** Results from the ascitic fluid

Investigation	Result
Microscopy, culture and sensitivity	Polymorphs 318 × 10^6^/L, lymphocytes 156 × 10^6^/L, other nucleated cells 132 × 10^6^/L, no organisms seen, no growth after 2 days.
Cytology	Cellular fluid consisting of mesothelial cells, histocytes including scanty multinucleated giant forms, polymorphs and lymphocytes. No malignant cells seen.
Acid fast bacilli stain	Negative
Albumin	27 g/L
Total protein	43 g/L
Creatinine	46 μmol/L
Amylase	41 U/L

A multi-disciplinary approach formed the basis for the attending team, with the involvement of gastroenterologists, oncologists, infectious diseases specialists and medical microbiologists. An oesophago-gastro-duodenoscopy and colonoscopy were initially performed, and both were macroscopically normal. Random biopsies were obtained, and histology from the duodenum demonstrated Coeliac disease. With dietetics input, her nutrition was modulated with good response. Specialist medium cultures and blood test e.g. Quantiferon, breast ultrasound, mammography and a CT chest showed no abnormalities. Vaginal swabs and transvaginal ultrasound did not yield any abnormalities. Her intra-uterine device (IUD) was removed by the gynaecology team and this was sent for culture but did not demonstrate any abnormal growth.

On Day 8, the patient became febrile with a rise in inflammatory markers (white cell count 20.0 × 10^9^/L). No localizing symptoms or signs were apparent and a septic screen (including ascitic tap) did not demonstrate an infective focus. This episode resolved after a 5-day trial of intravenous antibiotics (amoxicillin, metronidazole and gentamicin).

**Figure 2:**
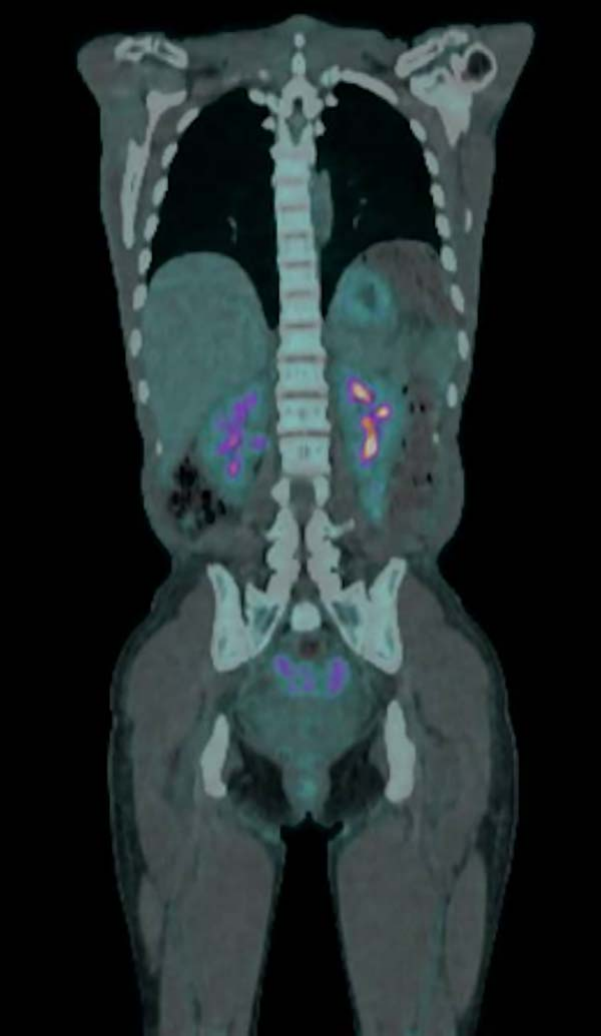
PET-CT.

A positron emission tomography-CT (PET-CT) scan demonstrated abnormal tracer uptake in the omentum, right lower abdomen quadrant and pouch of Douglas (Fig. 2). A subsequent diagnostic laparoscopy for targeted biopsies however yielded only inflammatory changes with no signs of malignant processes. Post-operatively, the ascites resolved spontaneously, and on out-patient review at 4 weeks repeat blood tests and all clinical parameters had returned to normal.

## DISCUSSION

Laparoscopic surgery has many recognized complications ranging from surgical site infection and shoulder tip pain through to bladder injury, bowel injury and abdominal abscess formation [3, 4]. Post-operative ascites, however, is veryrare.

A literature review returned only one other case of idiopathic ascites after laparoscopic appendicectomy [5], and a limited number of other cases from other laparoscopic procedures [6,7]. The authors hypothesized that the ascites could have been secondary to an inflammatory response to chemicals. Another case of ascites after lymphatic injury during laparoscopy was also reported [8]. Ascites after other laparoscopic procedures has also been observed. In these reported cases the time between laparoscopy and development of ascites ranged from 1 to 14 days. The case presented here differs, with the interval being considerably delayed at some 2 months after the procedure was performed.

While this case resulted in spontaneous resolution and a full recovery, an extensive work-up was required to exclude more common and more sinister pathologies, such as malignant and infective causes of ascites.

Although rare, idiopathic ascites after laparoscopy may occur and appears to have a good prognosis with spontaneous resolution. However, an extensive diagnostic work-up is critical to ensure patient safety and clinical recovery. While this case represents a very rare complication of laparoscopic appendicectomy, it illustrates an approach to this diagnostic dilemma and may guide clinicians on the potential natural history of this condition.
